# Emotional intelligence, perceived stress and academic performance of Sri Lankan medical undergraduates

**DOI:** 10.1186/s12909-017-0884-5

**Published:** 2017-02-20

**Authors:** P. Ranasinghe, W. S. Wathurapatha, Y. Mathangasinghe, G. Ponnamperuma

**Affiliations:** 1 Department of Pharmacology, Faculty of Medicine, Univeristy of Colombo, Colombo, Sri Lanka; 2 Department of Anatomy, Faculty of Medicine, University of Colombo, Colombo, Sri Lanka; 3 Department of Medical Education, Faculty of Medicine, University of Colombo, Colombo, Sri Lanka

**Keywords:** Emotional intelligence, Perceived stress, Academic performance, Medical students, Sri Lanka

## Abstract

**Background:**

Previous research has shown that higher Emotional Intelligence (EI) is associated with better academic and work performance. The present study intended to explore the relationship between EI, perceived stress and academic performance and associated factors among medical undergraduates.

**Methods:**

This descriptive cross-sectional research study was conducted among 471 medical undergraduates of 2nd, 4th and final years of University of Colombo, Sri Lanka. Students were rated on self administered Perceived Stress Scale (PSS) and Schutte Self-Report Emotional Intelligence Test (SEIT). Examination results were used as the dichotomous outcome variable in a logistic regression analysis.

**Results:**

Females had higher mean EI scores (*p* = 0.014). A positive correlation was found between the EI score and the number of extracurricular activities (*r* = 0.121, *p* = 0.008). Those who were satisfied regarding their choice to study medicine, and who were planning to do postgraduate studies had significantly higher EI scores and lower PSS scores (*p* <0.001). Among final year undergraduates, those who passed the Clinical Sciences examination in the first attempt had a higher EI score (*p* <0.001) and a lower PSS score (*p* <0.05). Results of the binary logistic-regression analysis in the entire study population indicated that female gender (OR:1.98) and being satisfied regarding their choice of the medical undergraduate programme (OR:3.69) were significantly associated with passing the examinations. However, PSS Score and engagement in extracurricular activities were not associated with ‘Examination Results’.

**Conclusions:**

Higher EI was associated with better academic performance amongst final year medical students. In addition a higher EI was observed in those who had a higher level of self satisfaction. Self-perceived stress was lower in those with a higher EI. Enhancing EI might help to improve academic performance among final year medical student and also help to reduce the stress levels and cultivate better coping during professional life in the future.

**Electronic supplementary material:**

The online version of this article (doi:10.1186/s12909-017-0884-5) contains supplementary material, which is available to authorized users.

## Background

Emotional intelligence (EI) refers to a collection of skills such as self-control, determination, self-motivation and sensitivity to the feelings of others [1]. Different scholars have defined and explained the concept of EI in terms of models consisting of various emotional skills. Gardner in his book “Frames of Mind” published in 1983 proposed that there was a wide spectrum of intelligences that was crucial for success in life. For example, interpersonal intelligence comprising of leadership, the ability to nurture relationships, the ability to resolve conflicts and the skills of social analysis, was one such intelligence described by Gardner [2]. Yale University psychologist Peter Salovey defined EI in 5 domains, namely, knowing one’s emotions, managing emotions, motivating oneself, recognizing emotions in others and handling relationships [3]. In general, EI refers to a persons’ ability to recognize and regulate emotions in oneself and in others [4]. During the last two decades this new dimension of intelligence has received much attention as being more responsible for professional success than the Intelligence Quotient (IQ), the traditionally used measure of intelligence [5, 6]. EI involves skills such as motivation and determination, which plays an important role in achieving goals. A review by Mayer, et al found that higher EI is correlated positively with, better social relationships in children and adults, higher academic achievement, better relationships during work performance and enhanced psychological well-being [7].

EI is gaining increasing recognition in the field of medicine, as being important for doctors and other health care personnel when dealing with patients. Better intrapersonal EI may also be relevant to the high-stress working environment which doctors are constantly working under and required to deal with [8]. Accreditation Council for Graduate Medical Education (ACGME) in USA has defined 6 core competencies of a medical graduate: patient care, professionalism, system based practice, interpersonal and communication skills, medical knowledge and practice based learning [9]. Most of the skills that are highlighted in the above competencies are components of EI [9]. Numerous studies have been conducted to assess the EI and its correlates among medical students and other health care professionals. Furthermore, previous research has shown that higher EI is associated with better academic performance [10–14], better clinical skills [15] and higher work performance [16].

Although emotions are known to be a universal phenomena, cultural factors can strongly influence the ways in which they are being experienced/perceived, conveyed, and controlled [17]. South Asia (SA), also known as the Indian sub-continent, is home to nearly 20% of the world population, who share similar cultural beliefs and practices. At present there are numerous studies on EI of medical undergraduates (UGs) and postgraduates (PGs) from SA, which has explored the relationships between EI, empathy [18, 19] and stress [20, 21]. Presently there is a scarcity of studies looking at the co-relation between academic performance and EI from SA.

**Fig. 1:**
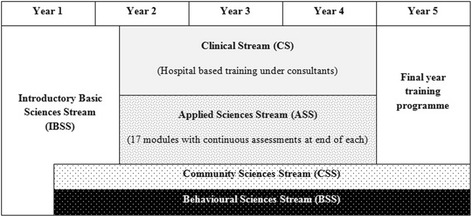
Present curriculum at Faculty of Medicine, University of Colombo

Sri Lanka is a rapidly developing nation in the SA region with a population of about 21 million. Established as the Colombo Medical School in 1870, the Faculty of Medicine, University of Colombo is the 2^nd^ oldest medical school in SA [22]. The medical undergraduate curriculum at the Faculty spans 5 years, where the initial 1½ years of pre-clinical teaching is followed by 3½ years of clinical teaching. The entire curriculum is subdivided into 5 main streams; Introductory Basic Sciences Stream (IBSS), Applied Sciences Stream (ApSS), Clinical Stream (CS), Community Sciences Stream (CSS) and Behavioural Sciences Stream (BSS). A pictorial representation of the present curriculum is given in Fig. 1.

The present study aims to evaluate the relationship between EI and academic performance among Sri Lankan medical undergraduate and study socio-demographic and other factors affecting EI. Furthermore, stress during medical schooling is an area increasingly being reported in the literature [23]. This can result in numerous negative effects on medical students and their training, including; impairment of class room functioning, disorders related to stress and performance deterioration. Hence, we also aim to explore the relationship between perceived stress, EI and academic performance.

## Methods

### Study population and sampling

This descriptive cross-sectional study was conducted at the Faculty of Medicine, University of Colombo, Sri Lanka from October-November 2015. All students who have completed the final examination of the IBSS (2^nd^ year UGs, *n* = 205), all modules of the ApSS (4^th^ year UGs, *n* = 193) and the End of CS examination (5^th^ year UGs, *n* = 198) were invited for the study (details of the above examinations are given below). Ethics approval for the study was obtained from the Ethics Review Committee, Faculty of Medicine, University of Colombo, Sri Lanka. The Study was conducted in accordance with the STROBE guidelines (Additional file 1).

### Study instrument and data collection

A self-administered questionnaire, consisting of three sections was used for data collection (Additional file 2). The questionnaire was distributed and data were collected after a lecture session in each group. Section A, evaluated socio-demographic details of the respondents (age, area of residence, gender, religion, ethnicity, monthly family income, parents level of education, parents employment status and students involvement in extra-curricular activities at the faculty [sports, societies and clubs, music, dancing, and etc]). In addition, the participants satisfaction regarding the choice to study medicine (satisfied/unsatisfied) and plans to do postgraduate studies was also evaluated. Section B, evaluated the EI of study participants using the validated 33-item self-assessment tool, Schutte Self-Report Emotional Intelligence Test [SEIT] [24]. Respondents rated themselves in each of the 33-items, according to a five point Likert scale (1 = strongly disagree to 5 = strongly agree). Scores can range from 33 to 165 with higher scores characterizing higher EI. Section C, consisted of the 10-item Perceived Stress Scale (PSS), the most commonly used questionnaire worldwide to assess the perceived stress levels of the individuals pertaining to different situations during the last month [25]. Each of the 10-items consists of a five point Likert scale (0 = never to 4 = very often), and the total score can vary from 0 to 40, where higher total scores indicates a higher level of perceived stress. Informed written consent was obtained from all study participants prior to data collection.

### Definitions

The IBSS spanning the initial 1½ years focuses on teaching the basic sciences of Anatomy, Physiology and Biochemistry. The students are evaluated at the end of the IBSS, with a barrier examination consisting of Multiple Choice Questions (MCQs), Structured Essay Questions (SEQs) and practical assessments in each of the 3 different basic sciences. Students who fail to achieve a minimum pass mark of 50 for any of the 3 subjects are considered to have failed the above examination. The students who pass are graded as follows, based on the cumulative mark of the IBSS examination; ‘First Class’ ≥ 70 marks, ‘Second Class – Upper division’ 65–69 marks, ‘Second Class – Lower division’ 60–64 marks and ‘Pass’ 50–59 marks. The ApSS comprises of 17 different modules based on different organ systems, while the CS is mainly focused on clinical and patient-oriented hospital training. Each module in the ApSS is evaluated with an end of the module assessment consisting of MCQs and SEQs. At the end of the ApSS the cumulative mark of all modules is used to grade students in a similar manner as described above.

Those who pass all 17 modules in the ApSS are allowed to progress in to the final year, where the teaching is mainly centered on five main specialties, namely Clinical Medicine, Obstetrics and Gynaecology, Paediatrics, Psychological Medicine and Surgery. At the end of the final year the students are evaluated at the End of CS examination, which consists of MCQs, SEQs, practical assessments and patient-based case discussions in each of the five specialties. Those who pass (>50% marks for each subject) are again graded in a similar manner as described above, using the cumulative marks of the End of CS examination. Hence, all the three streams described above (IBSS, ApSS and CS), uses a cumulative mark to grade the students.

### Statistical analysis

Data were analyzed with Statistical Package for Social Sciences (SPSS) software, version 14. Descriptive data are presented as %s or as mean ± SDs. Significance of associations was tested using Chi square for categorical variables and Student’s *t*-test for continuous variables. For the purpose of a logistic regression analysis, participants were classified into 2 sub-groups depending on examination results; (1) ‘Pass’ category: participants who have passed the CS, ApSS or IBSS examinations in the respective groups, and (2) ‘Fail’ category: those who have not passed the above examinations in the respective groups. A binary logistic-regression analysis was performed in all patients with ‘Examination Results’ as the dependent variable (0=’Fail’; 1=’Pass’) and EI score, PSS score, gender (0 = male; 1 = female), satisfaction about selecting medicine (0 = unsatisfied; 1 = satisfied) and extra-curricular activities (0 = no; 1 = yes) as the continuous or dichotomous independent variables and confounding factors. A backward elimination procedure was used to select the explanatory independent variables, where a p-value of 0.10 was considered as the cut-off for variable exclusion. In all analyses a p-value ≤ 0.005 was considered statistically significant (a Bonferroni type adjustment was made to reduce the Type I error in multiple analysis).

## Results

### Socio-demographic characteristics

Sample size was 471, the response rates of the 2^nd^ year, 4^th^ year and 5^th^ year UGs were; 89% (*n* = 183), 84% (*n* = 163) and 63% (*n* = 125) respectively. Males were 44% (*n* = 209) (2^nd^ year UG - 43.7% [*n* = 80], 4^th^ year UGs – 44.2% [*n* = 72], 5^th^ year UGs – 45.7% [*n* = 57]). Majority of the students (96%, *n* = 453) were Sri Lankans, while the remaining 18 students were from Bhutan. Among the Sri Lankan students, majority (27%, *n* = 127) were residing in the Colombo district and were of Sinhalese ethnicity (87%, *n* = 397). Majority of the student indicated that they were involved in extra-curricular activities (74.5%, *n* = 351). Forty four percent (*n* = 207) of the fathers of the students had higher education, while this was only 37.6% (*n* = 277) in the mothers. When parental employment status was considered among the study participants, 87.2% (*n* = 401) fathers and 54.3% (*n* = 254) mothers were employed. Monthly family income was above 60,000 Sri Lankan Rupees (US$ 414) in the majority (40.8%, *n* = 192) (Average family income in Sri Lanka is 45,878 Sri Lankan Rupees [US$ 316]).

### Emotional intelligence score

Mean EI scores of 2^nd^ year, 4^th^ year and 5^th^ year UGs were 122.4 ± 12.1, 122.7 ± 11.2 and 121.6 ± 13.1 respectively (*p* = NS). Mean EI score in females (123.5 ± 10.1) was higher than the mean EI score in males (120.7 ± 14.0) (*p* = 0.014, *d* = 0.22). The EI scores in students participating in extra-curricular activities (123.1 ± 11.8), was higher than in those not participating in extra-curricular activities (119.9 ± 12.4) (*p* = 0.013, *d* = 0.26). There was a significant positive correlation between the EI score and the number of extracurricular activities (*r* = 0.121, *p* = 0.008). Those who were satisfied regarding their choice to study medicine had a significantly higher EI score (123.8 ± 10.6), than those who were not satisfied (114.0 ± 17.4) (*p* <0.001, *d* = 0.68). Similarly, participants who were planning to do postgraduate studies, had a significantly higher EI score (123.6 ± 11.2) than those were not (117.6 ± 14.3) (*p* <0.001, *d* = 0.47).

Among 5^th^ year UG, those who passed the CS examination in the first attempt had a higher EI score (123.7 ± 9.6), than those who failed the CS examination (103.2 ± 22.7) (*p* <0.001, *d* = 1.18). This was not observed among the 2^nd^ year UG and 4^th^ year UG who passed/failed the IBSS examination and the ApSS in the first attempt. In the cohort who passed the IBSS (2^nd^ year UG), ApSS (4^th^ year UG) and CS examinations (5^th^ year UG), there was no significant difference in the EI scores in those who obtained a First/Second class and those who obtained a ‘Pass’. There was no significant difference in the EI scores between the different ethnicities, religions, areas of residence, family income levels, parents’ education and parents’ employment status (data not shown).

### Perceived stress score

The mean PSS scores of 2^nd^ year, 4^th^ year and 5^th^ year UGs were 19.9 ± 5.1, 19.6 ± 5.6 and 17.2 ± 5.1 respectively. The mean PSS of the 5^th^ year UGs was significantly lower the mean PSS of both 2^nd^ year UGs and 4^th^ year UGs (*p* <0.001). There was no significant difference in the mean PSS scores between the different genders, ethnicities, religions, areas of residence and PSS scores were not influenced by the engagement in extra-curricular activities, monthly family income, parents’ educations and employment status (data not shown). Those who were satisfied regarding their choice to study medicine had a significantly lower mean PSS score (18.4 ± .4.9), than those who were not satisfied (22.4 ± 6.2) (*p* <0.001, *d* = 0.71). Similarly, participants who were planning to do postgraduate studies, had a significantly lower PSS score (18.7 ± 5.2) than those were not (21.2 ± 5.7) (*p* <0.001, *d* = 0.46).

Among 5^th^ year UG, those who passed the CS examination in the first attempt had a lower PSS score (16.9 ± 4.9), than those who failed the CS examination (19.8 ± 4.9) (*p* <0.05, *d* = 0.59). However, this was not observed between those who passed or failed the ApSS among the 4^th^ year UGs and those who passed or failed the IBSS examination among the 2^nd^ year UGs. In the all three groups there was no significant difference in the mean PSS scores in those who obtained a First/Second class and those who obtained a ‘Pass’.

### Results of the regression analysis

Table 1 summarizes the results of the binary logistic-regression analysis. The overall model demonstrated statistical significance, the Cox & Snell R-Square and Nagelkerke R Square values were 0.086 and 0.127 respectively. The final results indicated that being satisfied regarding their choice of the medical undergraduate programme (OR: 3.69) and female gender (OR: 1.98) were the only significant factor associated with passing the examinations. However, EI Score, PSS Score and engagement in extra-curricular activities were not associated with ‘Examination Results’ (Table 1).

**Table 1:** Results of the binary logistic regression analysis

Risk factors	Odds ratio (95% CI)	*p* Value
Female gender	1.98 (1.05–1.57)	0.003
Satisfaction	3.69 (1.84–7.49)	<0.001
EI Score	1.31 (1.05–1.55)	0.043
PSS Score	1.01 (0.96–1.06)	0.642
Extra-curricular activities	1.40 (0.81–2.41)	0.224

## Discussion

To our knowledge, this is the first study which assessed the relationship between EI, perceived stress and academic performance in a large cohort of medical students from three different years of the undergraduate medical curriculum. Our results demonstrate that higher EI is associated with better academic performance amongst final year medical students. Similar results have been observed in other smaller studies conducted elsewhere, not only among medical students, but also among nursing students [10, 13, 26]. This finding is important because EI can be increased with deliberate practice and training, unlike IQ (Intelligence Quotient), which does not change significantly over a persons’ lifetime [26, 27]. Research on EI has shown that it is associated with more pro-social behaviors, improved empathy towards patients and a better patient care [10]. Furthermore, our results also demonstrate that the level of self satisfaction was significantly higher among medical students who had higher EI scores. EI is known to have a positive relationship with contentment and life satisfaction when adjusted for personality factors, which is another benefit of enhancing EI among medical undergraduates [28].

In the present cohort the EI scores in students who were involved in extra-curricular activities were higher and the EI scores were positively correlated with the number of extracurricular activities. Although this difference was not statistically significant, such an association has not been demonstrated previously among medical students. Further studies are required to explore such an association, as extra-curricular activities could be utilized to enhance EI if a causal relationship association is demonstrated. Involvement in extra-curricular activities might help to increase EI, by enhancing one’s capacity to be aware of and express emotions, and to handle interpersonal relationships. This association could also mean that an emotionally well balanced person can manage a number of activities together, in-spite of tight schedules. Multitasking is a core skill in all branches of medicine, especially in fields like emergency medicine [29].

The EI scores in all three groups were similar, and did not demonstrate any improvement from 1^st^ year to final year. Since the students in all three groups had similar socio-demographic characteristics, it can be assumed that the present undergraduate curriculum does not enhance or deteriorate students’ EI. Effective formal/informal training should be considered to improve students’ EI during the undergraduate training. Numerous research studies have demonstrated that the ability of a doctor to deliver safe and compassionate health care is influenced strongly by his/her EI [30]. The undergraduate curriculum needs to be sensitive to the requirement for EI based education, which may help to foster professionalism and communication skills among future doctors. Our results also demonstrated a gender difference in EI, where female students had a higher EI score than male students, a result observed collectively and also independently in all three groups. However, the association was not statically significant. Higher EI might also partially explain the association between female gender and educational performance observed in the regression analysis. However, the effect of gender on EI has been widely debated over the last few decades, with several studies supporting [31, 32] and an equal number refuting the association between female gender and higher EI [31, 33].

Better EI was associated with a lesser self-perceived stress score as measured by the PSS. Similar results have been observed in studies conducted elsewhere, where higher EI has been associated with reduced level of stress and better coping with stressors, not only amongst medical students, but also in nursing and other related health care professionals [20, 32, 34, 35]. Studies have shown that medical students experience significant psychological distress during their period of undergraduate training and that they have substantially higher levels of stress when compared to the normal population [36, 37]. Hence, if sufficient measures are adopted to improve EI among students, it would reduce the stress levels and cultivate better coping amongst the students during their training years in medical school and also help during their practice as professionals in the future. It is well known that healthcare workers with higher job satisfaction have lower stress levels [37]. A similar negative correlation has been observed among university students [38]. In the present cohort, the self-satisfied group of students had a significantly lower stress level.

The primary limitations of the present study are mainly related to the limitations of the study instruments used. It is argued that self-ratings of EI provides an indication of the respondents beliefs about their EI (perceived EI), rather than reflecting upon their actual capacity and tends to be positively biased [39]. Ratings from others (multi-rater or 360 degree evaluations) can serve an important role in providing insights into the extent to which others perceive certain behaviours and/or levels of performance [40]. Although such measures are useful in work-based environments, where numbers to be evaluated are relatively small, their usefulness in evaluating a large cohort is limited due to practicality. Common method variance (variance that is attributable to the measurement method rather than to the constructs the measures represent) caused by the study instrument is another limitation of the study, this is especially encountered in questionnaire based cross-sectional studies on attitude/behavioral constructs such as in the present study [41]. The PSS questionnaire evaluated the perceived stress levels during the previous month, since individual stress levels can be subjected to rapid fluctuations. Even then, recall bias might have affected self-reported stress levels. Furthermore, the cross-sectional design of our study limits the inference of causality for the factors identified as being associated with EI. Therefore, it is important to conduct prospective follow up studies to look for causality.

## Conclusions

Higher EI was associated with better academic performance amongst final year medical students. In addition a higher EI was observed in those who had a higher level of self satisfaction. Self-perceived stress was lower in those with a higher EI. Enhancing EI might help to improve academic performance among final year medical students and also might help to reduce the stress levels and cultivate better coping during professional life in the future.

## Additional files

Additional file 1: STROBE Checklist (Contains the details of the STROBE Checklist items and relevant page numbers of the manuscript). (DOC 88 kb)
Additional file 2: Questionnaire (Contains the study instrument used for data collection). (DOC 105 kb)

## Abbreviations

ACGME: Accreditation council for graduate medical education
ApSS: Applied sciences stream
BSS: Behavioural sciences stream
CS: Clinical stream
CSS: Community sciences stream
EI: Emotional intelligence
IBSS: Introductory basic sciences stream
IQ: Intelligence quotient
MCQ: Multiple choice questions
OR: Odds ratio
PG: Postgraduates
PSS: Perceived stress scale
SEIT: Schutte self-report emotional intelligence test
SEQ: Structured essay questions
SPSS: Statistical package for social sciences
UG: Undergraduates
