# Supplementary material for: Emotional intelligence, perceived stress and academic performance of Sri Lankan medical undergraduates
Source: BMC Med Educ. 2017 Feb 20;17:41. doi: 10.1186/s12909-017-0884-5 (PMC5319135; doi:10.1186/s12909-017-0884-5)
Supplement: Additional file 2: — Questionnaire (Contains the study instrument used for data collection). (DOC 105 kb) [file 12909_2017_884_MOESM2_ESM.doc]

**Questionnaire**

1. Your MFC number ………………………………………
2. Area of residence District……………………Town……………………………

**Put a cross ‘×’ in the relevant box**

1. Gender Male Female
2. Religion Buddhism Hinduism

Christianity Islam

Other (please specify)…………………………

1. Ethnicity Sinhalese Tamil

Muslim Burgher

Other (please specify)…………………………

1. Your Parents’ educational level?

|  | Mother | Father |
| --- | --- | --- |
| Below O/L |  |  |
| Up to O/L |  |  |
| Up to A/L |  |  |
| Higher education |  |  |
| Other |  |  |

1. Your parents’ employment status?

Mother employed unemployed

Father employed unemployed

If employed please mention the occupation

Mother ………………………… Father……………………………….

1. Your family monthly Income?

< Rs. 20 000/= Rs.40 00/= - 60 000/=

Rs. 20 000/= - 40 000/= > Rs. 60 000/=

1. Have you engaged in any extracurricular activity in the faculty? Yes / No

If ‘Yes’ , what is it? Clubs/ Societies Sports Music

Dancing Drama

Other (please specify)……………………………

1. Below are a series of 33 statements. Indicate the extent to which each item applies to you by circling the corresponding number. There are no right or wrong answers. However, it is essential that your responses truly reflect your beliefs regarding the extent to which each item applies to you. You should not answer in a way that you think sounds good or acceptable. If you make a mistake simply cross it out and fill in the correct response.

|  | Strongly disagree | Disagree | Neither disagree nor agree | Agree | Strongly agree |
| --- | --- | --- | --- | --- | --- |
| 1. I know when to speak about my personal problems to others | 1 | 2 | 3 | 4 | 5 |
| 1. When I am faced with obstacles, I remember times I faced similar obstacles and overcame them. | 1 | 2 | 3 | 4 | 5 |
| 1. I expect that I will do well on most things I try | 1 | 2 | 3 | 4 | 5 |
| 1. Other people find it easy to confide in me | 1 | 2 | 3 | 4 | 5 |
| 1. I find it hard to understand the non-verbal messages of other people | 1 | 2 | 3 | 4 | 5 |
| 1. Some of the major events of my life have led me to re-evaluate what is important and not important | 1 | 2 | 3 | 4 | 5 |
| 1. When my mood changes, I see new possibilities | 1 | 2 | 3 | 4 | 5 |
| 1. Emotions are one of the things that make my life worth living | 1 | 2 | 3 | 4 | 5 |
| 1. I am aware of my emotions as I experience them | 1 | 2 | 3 | 4 | 5 |
| 1. I expect good things to happen | 1 | 2 | 3 | 4 | 5 |
| 1. I like to share my emotions with others | 1 | 2 | 3 | 4 | 5 |
| 1. When I experience a positive emotion, I know how to make it last | 1 | 2 | 3 | 4 | 5 |
| 1. I arrange events others enjoy. | 1 | 2 | 3 | 4 | 5 |
| 1. I seek out activities that make me happy. | 1 | 2 | 3 | 4 | 5 |
| 1. I am aware of the non-verbal messages I send to others | 1 | 2 | 3 | 4 | 5 |
| 1. I present myself in a way that makes a good impression on others | 1 | 2 | 3 | 4 | 5 |
| 1. When I am in a positive mood, solving problems is easy for me | 1 | 2 | 3 | 4 | 5 |
| 1. By looking at their facial expressions, I recognize the emotions people are experiencing. | 1 | 2 | 3 | 4 | 5 |
| 1. I know why my emotions change | 1 | 2 | 3 | 4 | 5 |
| 1. When I am in a positive mood, I am able to come up with new ideas | 1 | 2 | 3 | 4 | 5 |
| 1. I have control over my emotions | 1 | 2 | 3 | 4 | 5 |
| 1. I easily recognize my emotions as I experience them | 1 | 2 | 3 | 4 | 5 |
| 1. I motivate myself by imagining a good outcome to tasks I take on | 1 | 2 | 3 | 4 | 5 |
| 1. I compliment others when they have done something well | 1 | 2 | 3 | 4 | 5 |
| 1. I am aware of the non-verbal messages other people send | 1 | 2 | 3 | 4 | 5 |
| 1. When another person tells me about an important event in his or her life, I almost feel as though I have experienced this event myself | 1 | 2 | 3 | 4 | 5 |
| 1. When I feel a change in emotions, I tend to come up with new ideas | 1 | 2 | 3 | 4 | 5 |
| 1. When I am faced with a challenge, I give up because I believe I will fail | 1 | 2 | 3 | 4 | 5 |
| 1. I know what other people are feeling just by looking at them | 1 | 2 | 3 | 4 | 5 |
| 1. I help other people feel better when they are down | 1 | 2 | 3 | 4 | 5 |
| 1. I use good moods to help myself keep trying in the face of obstacles | 1 | 2 | 3 | 4 | 5 |
| 1. I can tell how people are feeling by listening to the tone of their voice | 1 | 2 | 3 | 4 | 5 |
| 1. It is difficult for me to understand why people feel the way they do | 1 | 2 | 3 | 4 | 5 |

1. Below mentioned questions ask you about your feelings and thoughts **during the last month**. In each case, indicate how often you felt or thought a certain way by circling corresponding number.

**0 = Never 1 = Almost Never 2 = Sometimes 3 = Fairly Often 4 = Very Ofte**n

| 1. In the last month, how often have you been upset because of something that happened unexpectedly? | 0 | 1 | 2 | 3 | 4 |
| --- | --- | --- | --- | --- | --- |
| 1. In the last month, how often have you felt that you were unable to control the important things in your life? | 0 | 1 | 2 | 3 | 4 |
| 1. In the last month, how often have you felt nervous and “stressed”? | 0 | 1 | 2 | 3 | 4 |
| 1. In the last month, how often have you felt confident about your ability to handle your personal problems? | 0 | 1 | 2 | 3 | 4 |
| 1. In the last month, how often have you felt that things were going your way? | 0 | 1 | 2 | 3 | 4 |
| 1. In the last month, how often have you found that you could not cope with all the things that you had to do? | 0 | 1 | 2 | 3 | 4 |
| 1. In the last month, how often have you been able to control irritations in your life? | 0 | 1 | 2 | 3 | 4 |
| 1. In the last month, how often have you felt that you were on top of things? | 0 | 1 | 2 | 3 | 4 |
| 1. In the last month, how often have you been angered because of things that were outside of your control? | 0 | 1 | 2 | 3 | 4 |
| 1. In the last month, how often have you felt difficulties were piling up so high that you could not overcome them? | 0 | 1 | 2 | 3 | 4 |

If you are a 2^nd^ Year Student Answer Questions 13-15 and go to Question 22

If you are a 4^th^ Year Student Answer Questions 16-17 and go to Question 22

If you are a 5^th^ Year Student Answer Questions 18-21 and go to Question 22

1. Did you pass all 3 subjects of Basic Sciences Stream at first attempt? Yes / No (If you answer is ‘No’ please go to question number 15)
2. What is your IBSS result?

First class Second Upper Second Lower Pass

1. Have you got any Distinctions? Yes / No

If ‘Yes’, for which subjects you have got Distinctions?

Anatomy Physiology Biochemistry

15. If your answer is “No” to question no: 12, how many subjects you had to repeat? …………

16. Did you pass all the module exams of Applied Sciences Stream at first attempt? Yes / No

17. If your answer to above question is “No” how many modules you had to repeat? ……………

18. Were you able to pass all the subjects of Clinical Sciences Stream at first attempt? Yes / No

19. If your answer to above question is ‘Yes’ what was your result?

First class Second upper Second lower Pass

20. If your answer to question no. 13 is ‘No’, how many subjects you had to repeat? …………...

21. What was your Cumulative MBBS result at first attempt?

First class Second upper Second lower Pass

Referred

22. Are you satisfied about the decision you made to learn medicine?

Very satisfied Unsatisfied

Satisfied Very Unsatisfied

Not sure

23. Do you have a plan of doing post graduate studies? Yes / No

24. Where do you stay currently?

At home Boarding place

Hostel At relative’s place

Other (please specify) ……………………………

Thank You.
